# Active Terahertz Chiral Metamaterials Based on Phase Transition of Vanadium Dioxide (VO_2_)

**DOI:** 10.1038/s41598-017-18472-x

**Published:** 2018-01-09

**Authors:** Shengxiang Wang, Lei Kang, Douglas H. Werner

**Affiliations:** 1 School of Electronic and Electrical Engineering, Wuhan Textile University, Wuhan, Hubei 430073 China; 2 Department of Electrical Engineering, The Pennsylvania State University, University Park, PA 16802 USA; 3 State Key Laboratory for Hubei New Textile Materials and Advanced Processing Technology, Wuhan Textile University, Wuhan, 430020 China

## Abstract

Compared with natural materials, chiral metamaterials have been demonstrated with orders of magnitude stronger chiroptical response, which provides the basis for applications such as ultracompact polarization components and plasmonic-enhanced biosensing. Terahertz chiral metamaterials that allow dynamic polarization control of terahertz waves are of great practical interest, but remain extremely rare. Here, we show that hybrid metamaterials integrated with vanadium dioxide (VO_2_) exhibiting phase transition can enable dynamically tunable chiroptical responses at terahertz frequencies. In particular, a circular dichroism of ~40° and a maximum polarization rotation of ~200°/λ are observed around 0.7 THz. Furthermore, our study also reveals that the chiroptical response from the proposed metamaterials is strongly dependent on the phase transition of VO_2_, leading to actively controllable polarization states of the transmitted terahertz waves. This work paves the way for the development of terahertz metadevices capable of enabling active polarization manipulation.

## Introduction

Chiroptical responses, such as circular dichroism (CD) and optical activity, observed in natural materials, in general reflect the corresponding structural symmetry inherent at the microscopic level. Accordingly, circular polarization has been utilized to generate, transmit and measure electromagnetic signals that carry important information during the light-matter interaction process. For example, CD spectrum spectroscopy, which is based on the difference in the absorption of circularly polarized light of opposite handedness, has been widely utilized in biosensing for the extraction of structural information of large biological molecules^1^. Moreover, chirality based manipulation of the polarization state of light has been exploited in various optical applications such as Faraday isolators^2^, which depend on the Faraday effect induced by an external magnetic field. However, due to the extremely weak chirality of naturally occurring media, bulk chiral materials are generally required to obtain a detectable chiroptical response.

Metamaterials have been shown to offer unprecedented flexibility in manipulating electromagnetic waves through design and tailoring of their subunit building blocks, *i.e*., the meta-atoms^3–5^. In particular, metamaterials comprised by meta-atoms lacking mirror symmetry have been implemented to realize a giant chiral response^6–23^. Such structures offer extensive potential for applications based on the far-field and near-field chiroptical effect, such as circular polarizers^10,19^, polarization rotators^20,22^ and plasmonic-enhanced biosensors^14,23,24^. Analogous to their counterparts for the manipulation of linearly polarized waves, conventional chiral metamaterials derive their resonant properties from the geometric arrangement of their meta-atoms and their constituent material composition. One consequence of this, however, is the fact that their chiroptical responses are generally fixed. To further functionalize metamaterials, hybridizations of active media (*e.g*., silicon, graphene and liquid crystals) with conventional metallic resonating components have been implemented to enable reconfigurability via external stimuli (see the recent review papers^24–26^). Recently, germanium antimony telluride (Ge_2_Sb_2_Te_5_ or “GST”), a type of phase change material, has been harnessed to facilitate thermally tunable chirality for chiral plasmonic nanostructures operating in the mid-infrared regime^27,28^.

Terahertz (THz) metamaterials, especially those endowed with tunability^29–47^, have proven to be highly effective in manipulating THz radiation in ways that are far superior to other natural media and therefore offer a tremendous potential for the creation of new and transformative THz devices. Among them, chiral metamaterials that provide the ability to dynamically control the polarization state of THz waves are of great importance for THz metadevices with sophisticated functionalities, but such examples remain rare. Photocarrier excitation enabled dynamic control of chirality has been reported in semiconductor based active THz metamaterials. In particular, Kanda and coauthors have demonstrated optical pumping induced THz optical activity in a semiconductor substrate which was masked by a two-dimensional array of chiral meta-atoms^33^. Zhang and coauthors have reported the handedness switching in three-dimensional THz chiral metamaterials in response to an external optical stimulus^36^. Zhou, *et al*., have verified that a variety of optically tunable chiral responses, including a chirality induced negative index of refraction, can be produced by a gold/silicon bilayer conjugate chiral microstrucuture^38^. Moreover, Lv, *et al*., have demonstrated a multiband background-free THz switch in a silicon filled bilayered chiral metamaterial^45^. Nevertheless, THz metamaterials capable of producing strong chiroptical responses that can be dynamically tuned over a large scale are highly sought after.

Experiencing atomic level deformation, phase change materials (PCMs) are capable of offering drastic variations in material properties over a broad spectral range during the phase transition. As a representative of PCMs, vanadium dioxide (VO_2_), which undergoes an insulator-to-metal transition (IMT) near room temperature (*T*_IMT_ ~67 °C), has been intensively studied due to its potential applications in both electronic and optical devices. Recently, VO_2_ has been introduced into metamaterial systems to enable response modulation of various nano-^48–50^ and micro-structured systems^31,35,47^ to external stimuli, such as temperature and electric current. Lv and coauthors have theoretically reported the observation of thermally tunable linear polarization conversion in a THz metamaterial composed of VO_2_-loaded twisted E-shaped metallic resonators^47^. We emphasize that besides an approximately three orders of magnitude increase in the THz conductivity (σ_1_) during IMT, the reported data^40^ indicates that σ_1_ of VO_2_ in its metallic state can be as high as 4 × 10^3^ Ω^−1^cm^−1^, which is roughly one order of magnitude lower than that of gold. Consequently, VO_2_ is able to support a strong THz resonance that can be exploited to achieve highly tunable THz devices. Recently, the temperature-tuned transmission of THz waves has been observed in metamaterials consisting of VO_2_ cut-wires^51^. We have also shown that the integration of VO_2_ structures with conventional metallic resonating components can enable a class of highly tunable THz metamaterials^52^.

In this work, we propose and demonstrate giant and actively controllable chiroptical responses from a VO_2_/gold twisted fishnet metamaterial. With VO_2_ in its metallic state, simulation results reveal a circular dichroism of ∼40° in the absolute value and a maximum polarization rotation of ∼200°/λ around 0.7 THz. Furthermore, we report highly-tunable chiroptical responses that are enabled by the VO_2_ phase transition. In particular, strong polarization state manipulation of a linearly polarized incident wave is identified, including the continuously modulated optical activity and ellipticity of the transmitted wave. Given the intensively studied phase-transition dynamics of VO_2_, we envision applications of the proposed hybrid metamaterials for ultrafast terahertz metadevices, such as circular polarizers and polarization modulators.

## Results

Since the two enantiomers of chiral objects exhibit identical scalar physical properties, remotely sensing the chiroptical response of an object provides a unique approach to enantioselectivity due to the fact that circularly polarized light (CPL) itself is chiral. Given the generally weak interaction between CPL and natural media, artificially structured materials have a long history of development with the goal of enhancing light-matter interaction and achieving stronger chiroptical effects. Owing to their resonant properties, the recent introduction of metamaterials offers unprecedented flexibility for realizing enormously strong chiral responses. Following the general design strategy, *i.e*., structures lacking mirror symmetry may lead to chirality, metamaterials composed of various chiral meta-atoms have been demonstrated to exhibit chiroptical responses orders of magnitude stronger than those found in natural media. Recently, as one of the compelling directions of metamaterials research, the comprehensive usage of nano-engineered metals has been reported to enable complex functionalities (*e.g*. Joule heating based modulation) in addition to optical resonance, which provides the basis for realizing ‘self-sufficient’ metadevices^50,53,54^. However, a close inspection of the reported chiral metamaterials reveals that most designs cannot support the additional functionalities because they typically consist of geometrically isolated units^13–20^. With a topologically continuous layered structure, twisted fishnet metamaterials are considered as good candidates for multifunctional chiral metadevices. By measuring the complex scattering parameters of a stack of twisted elliptical hole arrays, Wang, *et al*., have demonstrated large optical activity and circular dichroism at THz frequencies^55^. By implementing a scaled-down structure, Kang, *et al*., have demonstrated a photonic metamaterial with nonlinear chiral-selective electro-optic functionalities^54^. In addition, Rodrigues, *et al*., have reported intensity-dependent optical rotation in a similar twisted fishnet nanostructure^22^.

**Figure 1:**
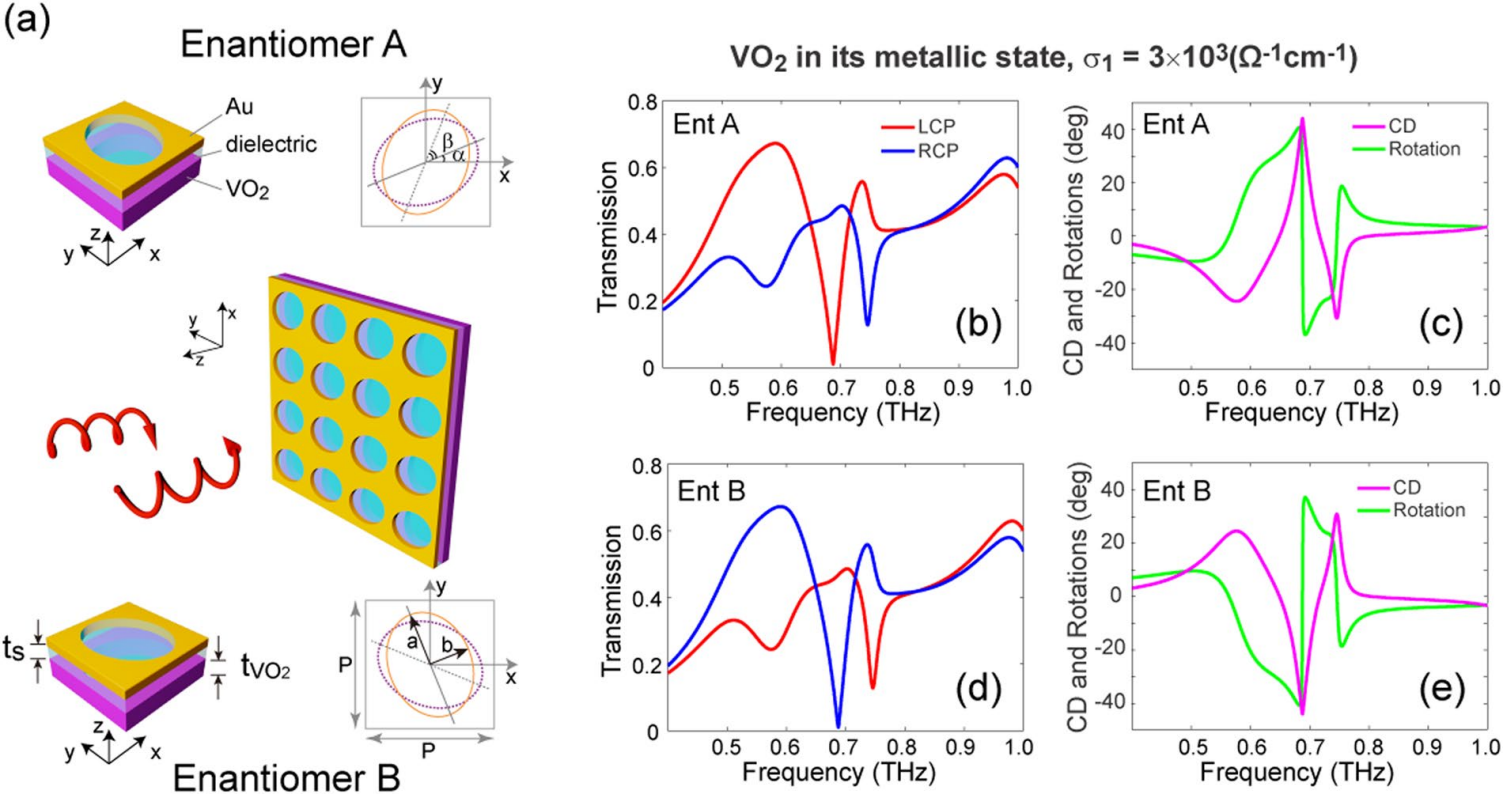
Hybrid terahertz chiral metamaterials based on VO_2_/metal twisted fishnet structures. (**a**) Schematic of the metamaterial which is composed of a gold and a VO_2_ layer perforated by identical elliptical holes and separated by an alumina spacer. The two enantiomers (mirror-image meta-atoms) are also depicted in (**a**). Geometrical parameters (all unit in μm): *P* = 130, *a* = 60, *b* = 40, *α* = 22.5°, *β* = 45°, *t*_VO2_ = 3, *t*_s_ = 42. For optimized performance, the metamaterial is embedded in a SiO_2_ dielectric matrix with a refractive index of 1.9. Transmission spectra of enantiomer A (**b**) and enantiomer B (**d**) for circularly polarized waves, when the VO_2_ is in its metallic state (σ_1_ = 3×10^3^ (Ω^−1^cm^−1^)). The corresponding circular dichroism (CD) and polarization rotation angle are shown in (**c**) and (**e**).

It has been shown that the giant chiral response from the twisted fishnet structures arises from the strong electromagnetic coupling between the two conductive layers under circular polarized illumination. Consequently, we start our design by adopting the THz conductivity of VO_2_ in its metallic state, *i.e*., σ_1_ = 3×10^3^ Ω^−1^cm^−1^ (see Methods for details)^40^. Figure 1(a) shows a three-dimensional illustration of the VO_2_/gold twisted fishnet metamaterial structure, along with the unit cell schematics of the two enantiomers which are mirror images of each other. A 3 μm-thick VO_2_ film and a 500 nm-thick gold film are perforated by identical elliptical holes (*a* = 60 and *b* = 40 μm) with angular offsets of *α* = 22.5° and *β* = 45° and separated by a dielectric spacer (*t*_*s*_ = 42 μm). It should be noted that in order to support a strong resonance in its metallic state, the VO_2_ film should not be thinner than the corresponding skin depth, which is ~1 μm around 1.0 THz. The unit cell is arranged in a two-dimensional square lattice with a lattice constant of 130 μm. The 2D projection plots of the two enantiomers clearly indicate the chiral symmetry of the meta-atoms. The transmission spectra of left- and right-handed circularly polarized (LCP and RCP) waves are illustrated in Fig. 1(b,d) for the two enantiomers, respectively. Handedness and enantiomer selectivity are immediately identified within three regions that exhibit pronounced chiral responses. In particular, for enantiomer A (B) a sharp transmission dip with near-zero transmission amplitude is observed around 0.70 THz for LCP (RCP) illumination. In addition, for circular polarization of the opposite handedness (*i.e*., RCP in Fig. 1(b) and LCP in Fig. 1(b)), two resonances with conspicuous spectral features can be identified around 0.58 and 0.75 THz. Interestingly, a pure linear polarization rotation is expected around the three critical frequencies, *i.e*., 0.65, 0.72 and 0.82 THz, where the relatively high transmission amplitude of the LPC and RCP waves are balanced. We note that the polarization rotation capability at multiple frequencies can be beneficial for multi-channel THz polarization components. To better illustrate the observed chiroptical phenomena, the corresponding circular dichroism (CD) and optical rotatory dispersion (ORD) are depicted in Fig. 1(c,e). In particular, CD and ORD are defined as CD = *tan* ^−1^[(*t*_*R*_ − *t*_*L*_)/(*t*_*R*_ + *t*_*L*_)] and *ORD* = 0.5[*arg*(*t*_*R*_) − *arg*(*t*_*L*_)], where *t*_*R*_ and *t*_*L*_ denote the complex transmission coefficients of RCP and LCP waves, while *arg* represents the phase angle. It can be seen that a maximum value of CD as large as ~44° (~−44°) is achieved in enantiomer A (B) around the excited resonance of the LCP (RCP) wave. Moreover, for the enantiomer A (B), ORD reaches ~30°, −27° and 6° (~−30°, 27° and −6°) at the three critical frequencies mentioned above. Considering the subwavelength thickness (~λ/9) of the metamaterial, a maximum polarization rotation of ~240°/λ around 0.7 THz is expected.

**Figure 2:**
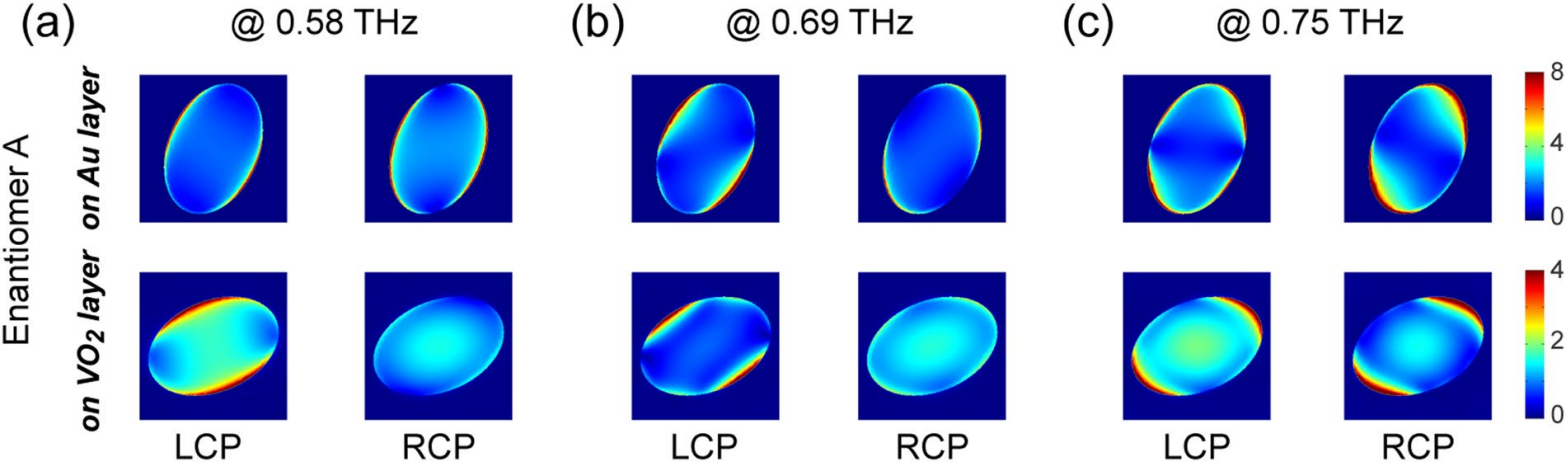
Mode analysis of chiral resonances. (**a**–**c**) The magnitude of the electric field (normalized to that of the incident field) at the resonance frequencies, when enantiomer A is illuminated by LCP and RCP waves. The cuts of the field distribution are through the middle of the VO_2_ and Au layers.

The chiral-selective transmission spectra shown in Fig. 1 indicate the distinct resonance modes corresponding to the LCP and RCP waves. To better illustrate the underlying physical mechanism of these chiral resonances, the cuts of the electric field distribution through the middle of the VO_2_ and Au layers for enantiomer A are shown in Fig. 2. Clearly, the handedness-selective resonance modes can be identified at each resonance frequency. In addition, for a circularly polarized (*e.g*. LCP) wave, the explicit field distribution at different resonances reflects the dispersion characteristic of the chiral metamaterial. Furthermore, referring to the CD spectra shown in Fig. 1(c,e), it is evident that the stronger field confinement in the central area of the holes on the exit layer (second row of Fig. 2) corresponds to the higher transmission for a certain handedness of CPL.

**Figure 3:**
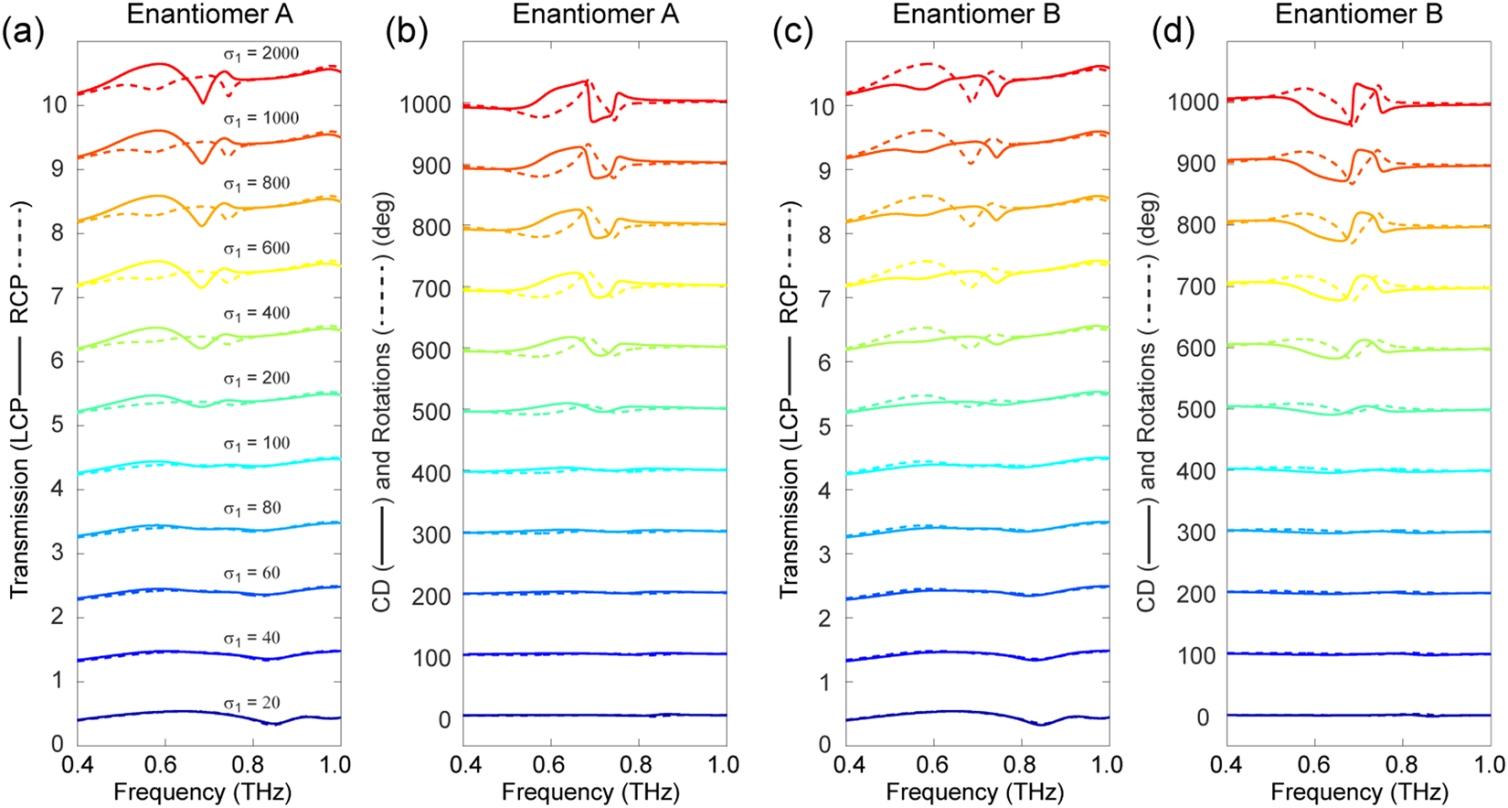
Phase transition of VO_2_ enabled tunable chiral responses. Simulated transmission spectra of enantiomer A (**a**) and enantiomer B (**c**) under LCP and RCP illumination for a series of VO_2_ conductivity (σ_1_ (unit in Ω^−1^cm^−1^)) values. The corresponding σ_1_ dependent CD and ORD of enantiomer A (**b**) and enantiomer B (**d**). For clarity, an offset has been added to the various curves in each plot.

**Figure 4:**
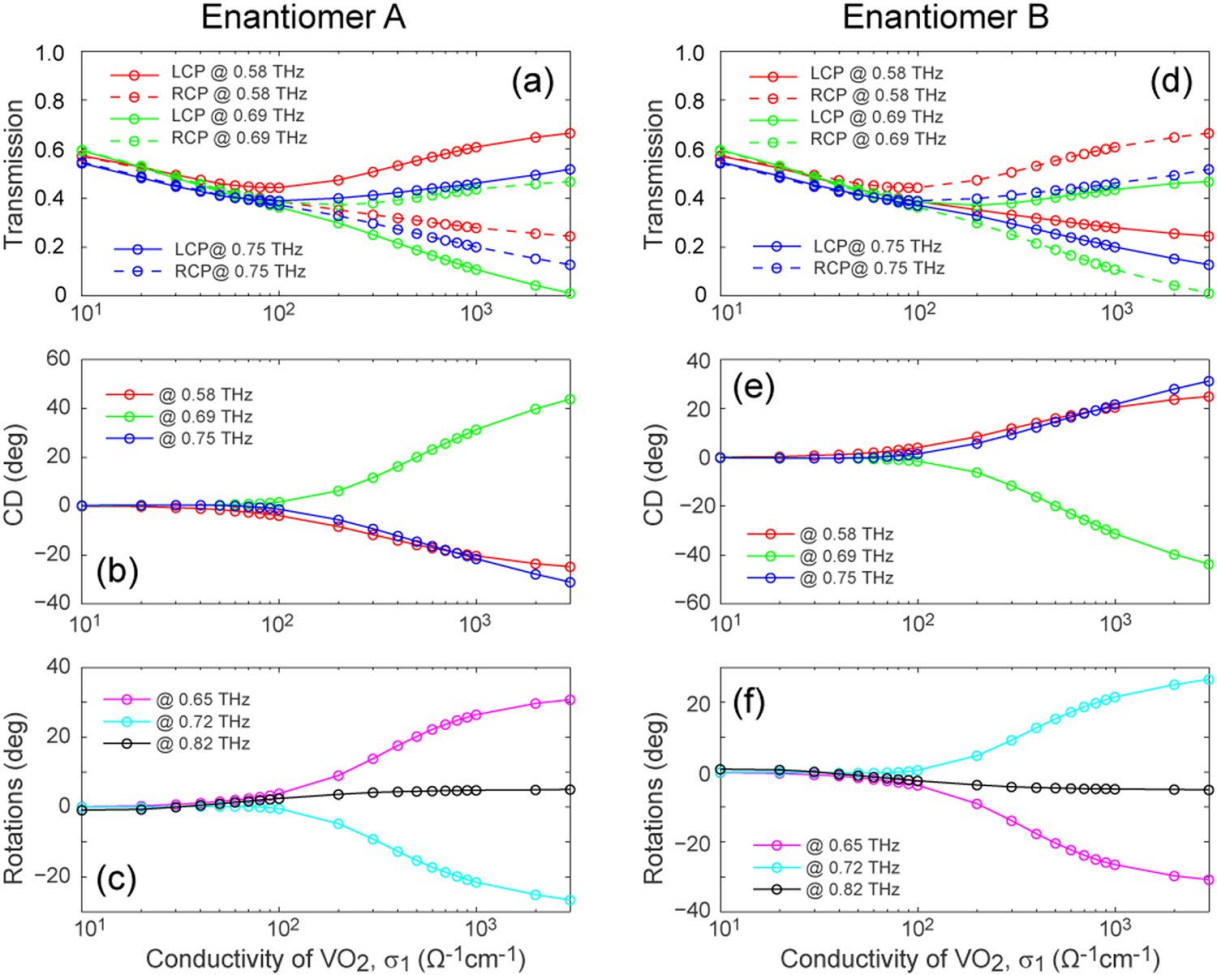
Chiroptical responses as a function of the THz conductivity of VO_2_. Simulated chiroptical responses at the critical frequencies from (**a**–**c**) enantiomer A and (**d**–**f**) enantiomer B for a series of VO_2_ conductivity values.

**Figure 5:**
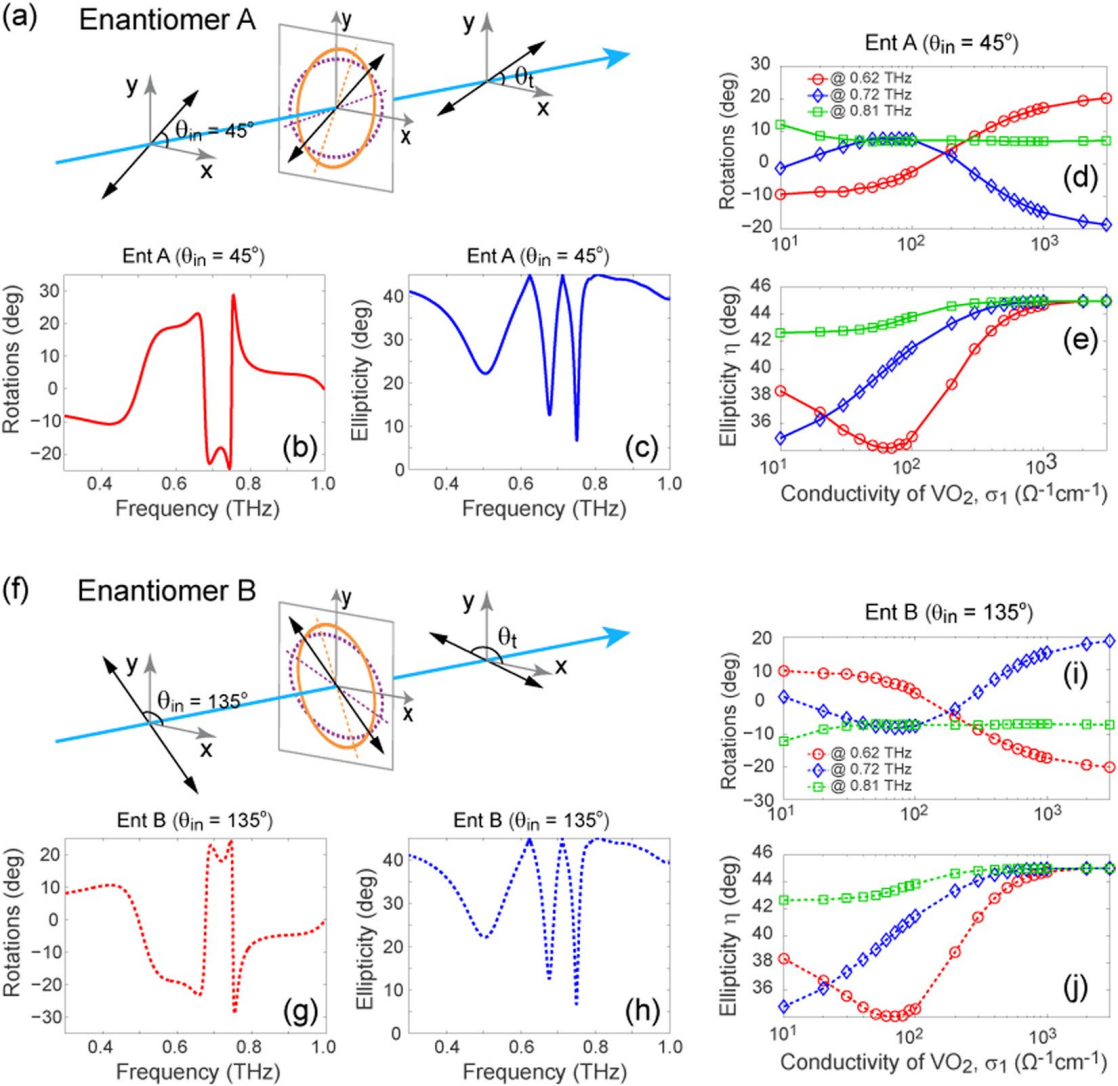
Optical rotatory of the VO_2_/metal twisted fishnet metamaterials. Schematic representation of the polarization rotation effect under consideration, in which a linearly polarized THz wave is normally incident on (**a**) enantiomer A (*θ*_in_ = 45°) and (**f**) enantiomer B (*θ*_in_ = 135°). The polarization azimuth rotation angle of the transmitted wave can be obtained from *θ*_t_ and *θ*_in_, *i.e*., rotation = *θ*_t_–*θ*_in_. The optical rotatory ((**b**) and (**g**)) and ellipticity ((**c**) and (**h**)) dispersion of enantiomer A and enantiomer B, when the VO_2_ is in its metallic state (σ_1_ = 3×10^3^ Ω^−1^cm^−1^). The rotation angle and ellipticity of the transmitted wave with a linear polarization transmitting through enantiomer A ((**d**) and (**e**)) and enantiomer B ((**i**) and (**j**)), for a series of VO_2_ conductivity values.

It is worth noting that, in sharp contrast to the methodologies previously developed and applied to hybrid metamaterials for achieving tunable responses to external stimuli, the active chiral metamaterials proposed here are based upon the highly tunable THz resonances realized by micro-structured VO_2_ experiencing a dramatic change in conductivity during the IMT. We note that the response of patterned VO_2_ film has been previously studied in the optical regime, for instance in ref.^48^. However, the corresponding structured VO_2_ in its metallic phase cannot support a strong resonance due to the rather high loss exhibited at optical frequencies. Consequently, the observed resonance tuning of the metallic structures can be regarded as a result of the variable index provided by the VO_2_ when in a different material phase. In our work, the resonance arising from the coupling between the structured VO_2_ film and the perforated gold layer gives rise to the strong chiral response. Figure 3 illustrated the simulated chiroptical responses of the metamaterial for a series of VO_2_ conductivity values. First of all, when the VO_2_ is nearly in its insulator state (σ_1_ = 20 Ω^−1^cm^−1^), the metamaterial manifests weak chirality, *i.e*., almost no difference in the transmission spectra of LCP and RCP waves (as shown in Fig. 3(a,c)) is found within the frequency range of interest. In addition, a continuous tuning of the systems’ chiroptical response is evident when σ_1_ increases before saturation. In particular, obvious chiral responses start to emerge in the three regions discussed above when σ_1_ is greater than 200 Ω^−1^cm^−1^. Furthermore, a comparison between the results of enantiomer A and B shown in Fig. 3 clearly reveals the enantiomer-selectivity of the metamaterial. To better display the phase transition enabled chiral responsive tuning effect, semi-log plots of the σ_1_-dependent transmission, CD and ORD at the critical frequencies are presented in Fig. 4 for both enantiomers. As depicted in Fig. 4(a,d)), for enantiomer A (B), the transmission of RCP (LCP) waves at 0.58 and 0.75 THz as well as that of LCP (RCP) waves at 0.69 THz show a nearly linear decrease in the semi-log plots. On the other hand, the corresponding transmission of circular polarizations of the opposite handedness increase when σ_1_ is greater than 100 Ω^−1^cm^−1^. These handedness- and enantiomer-selective behaviors, which are more clearly illustrated in Fig. 5(b,e), give rise to the distinct σ_1_ dependent CD characteristics. The ORD results shown in Fig. 4(c,f) portray the linear polarization rotation capability of the metamaterial, which is governed by σ_1._

Optical activity (also referred to as optical rotation) represents the ability of certain materials to rotate the polarization plane of a linearly polarized wave. Different from the Faraday effect (*i.e*., magnetic optical rotation), the strong optical activity observed in chiral metamaterials does not require the application of an external magnetic field and will potentially find applications in polarization manipulating components, such as ultracompact polarization rotators. Therefore, hereinafter we focus on the actively controllable optical rotation properties of the proposed metamaterial. It should be noted that Wang and coauthors^55^ have reported large THz optical activity of a similar gold based twisted fishnet structure, while, however, the corresponding transmission was rather low. As the results shown in Figs 1 and 2 implied, the proposed hybrid chiral metamaterial may exhibit strong polarization rotation power with fairly high transmission and, more importantly, the corresponding characteristic optical activity can be dynamically modulated over a large scale.

It has been experimentally verified that a linearly polarized wave with its polarization plane parallel to the direction where the two perforated ellipses intersect most closely experiences the strongest optical activity^22^. This incident polarization angle dependent behavior arises from the anisotropic property of the structure^20^ and can be eliminated by increasing the corresponding rotational symmetry. Accordingly, as a certain linearly polarized wave travels through the structure having low rotational symmetry, the actual optical rotation it undergoes may deviate from the ORD results discussed above. Consequently, without loss of generality, the input polarization angle (*θ*_in_) for enantiomer A and enantiomer B in our study is chosen to be 45° and 135°, respectively, as depicted in Fig. 5(a,f). Thereby, the polarization rotation angle can be obtained from the difference between *θ*_t_ and *θ*_in_, where *θ*_t_ denotes the angle between the long axis of the polarization ellipse of the transmitted wave and the *x* axis. Figure 5(b,g) illustrate the dispersion of the polarization rotation angle for a 45° and 135° linearly polarized THz wave, respectively, as they transmit through the enantiomeric metamaterials with VO_2_ in its metallic state. To obtain a complete picture of the polarization state of the transmitted waves, the corresponding ellipticity, defined as $\eta ={\tan }^{-1}((|{E}_{a}|-|{E}_{b}|)/(|{E}_{a}|+|{E}_{b}|)$, where $\,|{E}_{a}|$ and $|{E}_{b}|$ represent the semi-major and minor axis of the polarization ellipse of the electric field, is presented in Fig. 5(c,h). The minimum (maximum) value of η, *i.e*., 0° (45°), is obtained when the transmitted wave is circularly (linearly) polarized. It can be seen that the ellipticity reaches 45° at the frequencies of 0.62, 0.72 and 0.81 THz, indicating the linear polarization of the transmitted wave at these frequencies. As a result, in the following discussion we focus on these critical frequencies.

**Figure 6:**
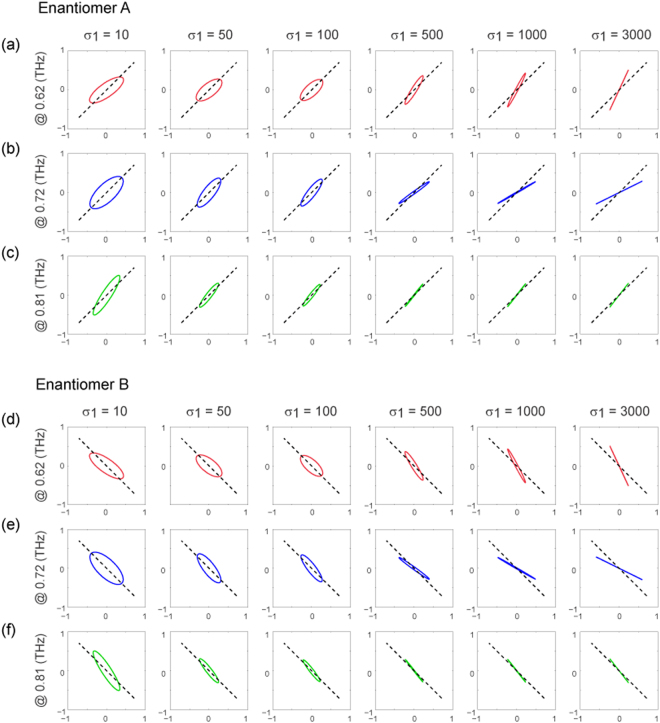
The polarization states for a linearly polarized incident THz wave transmitting through the hybrid chiral metamaterials. The projections of the field of the transmitted waves through enantiomer A ((**a**)–(**c**)) and enantiomer B ((**d**)–(**f**)) at 0.62, 0.72 and 0.81 THz, for a series of VO_2_ conductivity (σ_1_ (units in Ω^−1^cm^−1^)) values. The input linear polarizations (as depicted in Fig. 5(a,f)) are denoted by the dashed black line in the polarization graphics. The frequencies under consideration correspond to those where the linear polarization preservation occurs, as indicated by Fig. 5(c,h).

The polarization rotation angle and ellipticity as a function of the VO_2_ conductivity are presented on semi-log plots in Fig. 5(d,e) for enantiomer A and in Fig. 5(i,j) for enantiomer B. As expected, when σ_1_ increases the rotation angle of enantiomer B precisely exhibits opposite behavior compared to that of enantiomer A, once again confirming the enantiomeric characteristic of the metamaterials. Interestingly, for enantiomer A with VO_2_ in its insulator state (σ_1_ = 10 Ω^−1^cm^−1^), a polarization rotation angle of ~−10° and ~10° is observed at 0.62 and 0.81 THz, respectively. It should be noted that, as discussed above, this observation, which deviates from the results shown in Fig. 2(b) and Fig. 3(c), can be attributed to the anisotropic properties of the proposed metamaterial. Nevertheless, given the continuous increase of the rotation angle at 0.62 THz with σ_1_, this characteristic may in fact result in a corresponding polarization rotation modulation as large as ~30° (from ~−10° to ~20°). Another interesting observation is that the rotation angle at 0.72 THz increases from ~0° to ~7° when σ_1_ increases in the range from 10 Ω^−1^cm^−1^ to 100 Ω^−1^cm^−1^, while it decreases dramatically to ~−20° when σ_1_ approaches 3×10^3^ Ω^−1^cm^−1^. In other words, an optical activity switching can be achieved by controlling the phase transition of VO_2_. On the other hand, the change in σ_1_ may also tune the ellipticity of the transmitted waves, as displayed in Fig. 5(e) which is, as expected, identical to Fig. 5(j) for enantiomer B. For a better understanding of the evolution of the polarization state for a linearly polarized wave transmitting through the proposed metamaterials, the projections of the field of the transmitted waves are provided in Fig. 6. It can be seen that, for both enantiomers, a continuous and dramatic manipulation of the polarization state is evident for an incident THz wave at all three frequencies of interest.

## Discussion

Besides direct thermal control^56^ and optical pumping^57^, electric current and field-effect-induced phase transition in VO_2_ have also been reported^58,59^. Despite controversy over their microscopic origins, field-effect based transition is considered to have the potential to achieve VO_2_ property modulation at the subpicosecond level^58^. On the other hand, electrically controlled active metamaterials are of crucial importance due to their potential for a wide range of practical applications. In this respect, VO_2_ integrated hybrid metamaterials that exhibit electrically triggered active responses have attracted great attention. For instance, by integrating split ring resonators (SRRs) and VO_2_ thin films, Driscoll, *et al*., have demonstrated electrically controlled memory effects at THz frequencies^31^. Goldflam, *et al*., have reported electrical signal controlled spatial gradient tuning in a THz metamaterial consisting of an array of SRRs located on a VO_2_ layer^34^. Liu and coauthors demonstrated a VO_2_ integrated photonic metamaterial for electrically triggered multifunctional control^50^. To this end, as we discussed above, the topologically continuous structure of the proposed metamaterial provides the possibility of achieving response modulations through electrically induced phase transition in VO_2_. Furthermore, it is worth noting that, as a classical transition metal oxide, VO_2_ exhibits hysteresis behavior during its IMT. Therefore, the chiroptical responses produced by the proposed chiral metamaterials are expected to exhibit hysteretic characteristics, which means that, for example, the corresponding optical activity observed would be system history dependent. Taking into account this hysteretic behavior, the proposed metamaterial is expected to manifest a certain memory effect and, compared with the memory process observed in other metamaterial systems^31,50^, its chiral-selective properties may provide more degrees of freedom for designing metadevices with electro-optic information processing and storage capabilities. Moreover, given the Drude-type dispersion model of metallic-phase VO_2_ in the THz regime, the actively controllable response of the proposed hybrid metamaterial can be geometrically scaled to other frequencies.

To summarize, we have demonstrated that hybrid metamaterials based on VO_2_/metal twisted fishnet structures can enable dynamically tunable chiroptical responses at terahertz frequencies. Simulations show that, with VO_2_ in its metallic phase, a circular dichroism of ~44° and a maximum polarization rotation of ~240°/λ can be achieved around 0.7 THz. More importantly, we found that the chiroptical responses, such as circular dichroism and optical activity from the hybrid metamaterials are closely dependent on the phase transition related THz conductivity variation of VO_2_. Considering the hysteresis and ultrafast dynamics characteristics observed during the phase transition of VO_2_, we envision that the proposed hybrid chiral metamaterials may potentially enable chiroptical platforms for complicated manipulation of THz radiation.

## Methods

It has been experimentally verified that the permittivity of VO_2_ in the THz region can be described by the following Drude model: $\varepsilon (\omega )={\varepsilon }_{\infty }-\frac{{({\omega }_{{\rm{p}}}({\sigma }_{1}))}^{2}}{{\omega }^{2}+i\gamma \omega }$, where *ε*_∞_ is the permittivity at high frequency, *ω*_P_(*σ*_1_) is the conductivity (the imaginary part of the complex conductivity σ_2_ is assumed to be 0) dependent plasmon frequency and *γ* is the collision frequency^60^. A close examination reveals that both ${\omega }_{{\rm{p}}}^{2}$ and *σ*_1_ are proportional to the free carrier density and, as a result, the plasmon frequency at ${\sigma }_{1}\text{'}$ can be approximately expressed as ${\omega }_{{\rm{p}}}^{2}({\sigma }_{1}\text{'})=(\frac{{\sigma }_{1}\text{'}}{{\sigma }_{1}})\,{\omega }_{{\rm{p}}}^{2}({\sigma }_{1})$. By fitting the reported experimental data^40^, we have determined that when *σ*_1_ = 3 × 10^3^ Ω^−1^cm^−1^, it follows that *ω*_p_ = 1.40 × 10^15^ rad/s, while *γ* = 5.75 × 10^13^ rad/s is assumed to be independent of *σ*_1_. We note that these Drude model parameters are in good agreement with those reported by other groups^60^.

Full-wave simulations were implemented using the commercial finite integration package CST Microwave Studio. A unit cell of the investigated structure was simulated by employing periodic boundary conditions. The four transmission coefficients of circularly polarized waves, *i.e*., *t*_RR_, *t*_LR_, *t*_RL_ and *t*_LL_ can be expressed as a function of four linear transmission coefficients of the chiral medium, *i.e*., *t*_*xx*_, *t*_*yx*_, *t*_*xy*_ and *t*_*yy*_, such that ${t}_{{\rm{RR}}}=\frac{1}{2}[({t}_{xx}+{t}_{yy})+i({t}_{xy}-{t}_{yx})]$, ${t}_{{\rm{LR}}}=\frac{1}{2}[({t}_{xx}-{t}_{yy})+i({t}_{xy}+{t}_{yx})]$, ${t}_{{\rm{RL}}}=\frac{1}{2}[({t}_{xx}-{t}_{yy})-i({t}_{xy}+{t}_{yx})]$ and ${t}_{{\rm{LL}}}=\frac{1}{2}[({t}_{xx}+{t}_{yy})-i({t}_{xy}-{t}_{yx})]$. In addition, the polarization state for a linearly polarized wave transmitting through the metamaterials can be obtained from the total electric field of the transmitted waves, *i.e*., ${t}_{x}^{{\rm{total}}}={t}_{xx}\,\cos \,{\theta }_{in}+{t}_{xy}\,\sin \,{\theta }_{in}$ and ${t}_{y}^{{\rm{total}}}={t}_{yx}\,\cos \,{\theta }_{in}+{t}_{yy}\,\sin \,{\theta }_{in}$.

### Data availability

The data that support the findings of this study are available from the corresponding author on request.

## References

[1] Berova, N., Nakanishi, K. & Woody, R. W. Circular Dichroism: Principles and Applications. Wiley-VCH (2000).

[2] Saleh, B. E. A. & Teich, M. C. Fundamentals of Photonics. Wiley-Interscience (2007).

[3] Shalaev VM (2007). Optical negative-index metamaterials. Nat. Photon..

[4] Soukoulis CM, Wegener M (2011). Past achievements and future challenges in the development of three-dimensional photonic metamaterials. Nat. Photon..

[5] Liu YM, Zhang X (2011). Metamaterials: A new frontier of science and technology. Chem. Soc. Rev..

[6] Rogacheva AV (2007). Giant gyrotropy due to electromagnetic-field coupling in a bilayered chiral structure. Phys. Rev. Lett..

[7] Decker M (2007). Circular dichroism of planar chiral magnetic metamaterials. Opt. Lett..

[8] Plum E (2007). Giant optical gyrotropy due to electromagnetic coupling. Appl. Phys. Lett..

[9] Zhang S (2009). Negative refractive index in chiral metamaterials. Phys. Rev. Lett..

[10] Gansel JK (2009). Gold helix photonic metamaterial as broadband circular polarizer. Science.

[11] Zhou JF (2009). Negative refractive index due to chirality. Phys. Rev. B.

[12] Liu N (2009). Stereometamaterials. Nat. Photon..

[13] Decker M (2010). Twisted split-ring-resonator photonic metamaterial with huge optical activity. Opt. Lett..

[14] Hendry E (2010). Ultrasensitive detection and characterization of biomolecules using superchiral fields. Nat. Nanotechnol..

[15] Gao WS (2011). Circular dichroism in double-layer metallic crossed-gratings. J. Opt..

[16] Helgert C (2011). Chiral metamaterial composed of three-dimensional plasmonic nanostructures. Nano Lett..

[17] Dietrich K (2012). Circular dichroism from chiral nanomaterial fabricated by on-edge lithography. Adv. Mater..

[18] Zhao Y, Belkin MA, Alu A (2012). Twisted optical metamaterials for planarized ultrathin broadband circular polarizers. Nat. Commun..

[19] Thiel M (2013). Dip-in depletion optical lithography of three-dimensional chiral polarizers. Opt. Lett..

[20] Cui YH (2014). Giant chiral optical response from a twisted-arc metamaterial. Nano Lett..

[21] Plum E, Zheludev NI (2015). Chiral mirrors. Appl. Phys. Lett..

[22] Rodrigues SP (2017). Intensity-dependent modulation of optically active signals in a chiral metamaterial. Nat. Commun..

[23] Kang L, Ren Q, Werner DH (2017). Leveraging superchiral light for manipulation of optical chirality in the near-field of plasmonic metamaterials. ACS Photon..

[24] Zheludev NI, Kivshar YS (2012). From metamaterials to metadevices. Nat. Mater..

[25] Turpin JP (2014). Reconfigurable and tunable metamaterials: A review of the theory and applications. Int. J. Antennas Propag..

[26] Fan K, Padilla WJ (2015). Dynamic electromagnetic metamaterials. Mater. Today.

[27] Yin XH (2015). Active chiral plasmonics. Nano Lett..

[28] Tittl A (2015). A switchable mid-infrared plasmonic perfect absorber with multispectral thermal imaging capability. Adv. Mater..

[29] Chen H-T (2006). Active terahertz metamaterial devices. Nature.

[30] Chen H-T (2008). Experimental demonstration of frequency-agile terahertz metamaterials. Nat. Photon..

[31] Driscoll T (2008). Memory metamaterials. Science.

[32] Němec H (2009). Tunable terahertz metamaterials with negative permeability. Phys. Rev. B.

[33] Chen H-T (2009). A metamaterial solid-state terahertz phase modulator. Nat. Photon..

[34] Kanda N, Konishi K, Kuwata-Gonokami M (2009). Light-induced terahertz optical activity. Opt. Lett..

[35] Goldflam MD (2011). Reconfigurable gradient index using VO_2_ memory metamaterials. Appl. Phys. Lett..

[36] Zhang S (2012). Photoinduced handedness switching in terahertz chiral metamolecules. Nat. Commun..

[37] Gu J (2012). Active control of electromagnetically induced transparency analogue in terahertz metamaterials. Nat. Commun..

[38] Zhou J (2012). Terahertz chiral metamaterials with giant and dynamically tunable optical activity. Phys. Rev. B.

[39] Lee SH (2012). Switching terahertz waves with gate-controlled active graphene metamaterials. Nat. Mater..

[40] Liu M (2012). Terahertz-field-induced insulator-to-metal transition in vanadium dioxide metamaterial. Nature.

[41] Shrekenhamer D, Chen W-C, Padilla WJ (2013). Liquid crystal tunable metamaterial absorber. Phys. Rev. Lett..

[42] Fan K (2013). Optically tunable terahertz metamaterials on highly flexible substrates. IEEE Trans. Terahertz Sci. Technol..

[43] Chang C-L (2013). Tunable terahertz fishnet metamaterial. Appl. Phys. Lett..

[44] Li J (2013). Mechanically tunable terahertz metamaterials. Appl. Phys. Lett..

[45] Lv TT (2014). Optically controlled background-free terahertz switching in chiral metamaterial. Opt. Lett..

[46] Xu W-Z (2016). Electrically tunable terahertz metamaterials with embedded large-area transparent thin-film transistor arrays. Sci. Rep..

[47] Lv T-T (2016). Hybrid metamaterial switching for manipulating chirality based on VO_2_ phase transition. Sci. Rep..

[48] Dicken MJ (2009). Frequency tunable near-infrared metamaterials based on VO_2_ phase transition. Opt. Express.

[49] Seo M (2010). Active terahertz nanoantennas based on VO_2_ phase transition. Nano Lett..

[50] Liu (2016). Hybrid metamaterials for electrically triggered multifunctional control. Nat. Commun..

[51] Wen Q (2010). Terahertz metamaterials with VO_2_ cut-wires for thermal tunability. Appl. Phys. Lett..

[52] Wang S, Kang L, Werner DH (2017). Hybrid resonators and highly tunable terahertz metamaterials enabled by vanadium dioxide (VO_2_). Sci. Rep..

[53] Kang L (2014). Electrifying photonic metamaterials for tunable nonlinear optics. Nat. Commun..

[54] Kang L (2015). An active metamaterial platform for chiral responsive optoelectronics. Adv. Mater..

[55] Wang S (2013). Giant rotary power of a fishnet-like metamaterial. APL Mater..

[56] Qazilbash MM (2007). Mott transition in VO_2_ revealed by infrared spectroscopy and nano-imaging. Science.

[57] Cavalleri A (2001). Femtosecond structural dynamics in VO_2_ during an ultrafast solid-solid phase transition. Phys. Rev. Lett..

[58] Kim H-T (2004). Mechanism and observation of Mott transition in VO_2_-based two- and three-terminal devices. New J. Phys..

[59] Ruzmetov D (2010). Three-terminal field effect devices utilizing thin film vanadium oxide as the channel layer. J. Appl. Phys..

[60] Zhu Y (2012). Effect of substrate orientation on terahertz optical transmission through VO_2_ thin films and application to functional antireflection coatings. J. Opt. Soc. Am. B.

